# Proteome data associated with the leaf senescence in *Glycine max*

**DOI:** 10.1016/j.dib.2016.08.045

**Published:** 2016-08-28

**Authors:** Ravi Gupta, Su Ji Lee, Cheol Woo Min, So Wun Kim, Ki-Hun Park, Dong-Won Bae, Byong Won Lee, Ganesh Kumar Agrawal, Randeep Rakwal, Sun Tae Kim

**Affiliations:** a Department of Plant Bioscience, Life and Industry Convergence Research Institute, Pusan National University, Miryang 627-706, Republic of Korea; b Plant Molecular Biology and Biotechnology Research Center, Gyeongsang National University, Jinju, Republic of Korea; c Center for Research Facilities, Gyeongsang National University, Jinju, Republic of Korea; d Department of Functional Crops, National Institute of Crop Science (NICS), Rural Development Administration (RDA), Miryang 627-803, Republic of Korea; e Research Laboratory for Biotechnology and Biochemistry (RLABB), GPO Box 13265, Kathmandu, Nepal; f GRADE Academy Private Limited, Adarsh Nagar-13, Birgunj, Nepal; g Faculty of Health and Sport Sciences and Tsukuba International Academy for Sport Studies (TIAS), University of Tsukuba, 1-1-1 Tennodai, Tsukuba, Ibaraki 305-8577, Japan; h Global Research Center for Innovative Life Science, Peptide Drug Innovation, School of Pharmacy and Pharmaceutical Sciences, Hoshi University, 4-41 Ebara 2-chome, Shinagawa, Tokyo 142-8501, Japan

## Abstract

The data presented in this article are associated with the article “Coupling of gel-based 2-DE and 1-DE shotgun proteomics approaches to dig deep into the leaf senescence proteome of Glycine max” (R. Gupta, S.J. Lee, C.W. Min, S.W. Kim, K.-H. Park, D.-W. Bae, et al., 2016) [1]. Leaf senescence is one of the important aspects of the life cycle of a plant that leads to the recycling of nutrients from source to sink cells. To understand the leaf senescence-associated proteins, we used a combination of gel-based 2-DE and 1-DE shotgun proteomic approaches. Here, we display the 2-DE, Mass spectrometry, and Gene ontology data related with the leaf senescence in soybean [1].

**Specifications Table**

**Table:** 

Subject area	Biology
More specific subject area	Plant Science, Proteomics, Leaf Senescence
Type of data	Tables and figures
How data was acquired	Mass spectroscopy, MALDI-TOF/TOF-MS (ABI 4800, Applied Biosystems, Framingham, MA, USA) and UHPLC Dionex UltiMate^®^ 3000 (Thermo Fisher Scientific, USA) system coupled with QExactive^TM^ Orbitrap High-Resolution Mass Spectrometer (Thermo Fisher Scientific, USA)
Data format	Raw, analyzed
Experimental factors	Natural leaf senescence
Experimental features	Leaf senescence-associated proteins were identified
Data source location	Department of Functional Crop, National Institute of Crop Science (NICS), Rural Development Administration (RDA) at Miryang, South Korea (latitude 35N)
Data accessibility	Data are within this article

**Value of the data**

1. This data set depicts the comparative proteome analysis between two contrasting stages of leaf development, R3 (mature leaf) and R7 (senescent leaf).
2. A total of 1234 proteins were identified from R3 and R7 leaves using a combination of 2-DE and shot-gun proteomic approaches.
3. Data reported here deepen our understanding on leaf senescence at proteome level and could be used to develop senescence specific biomarker(s) in future.

## 1 Data

**Fig. 1:**
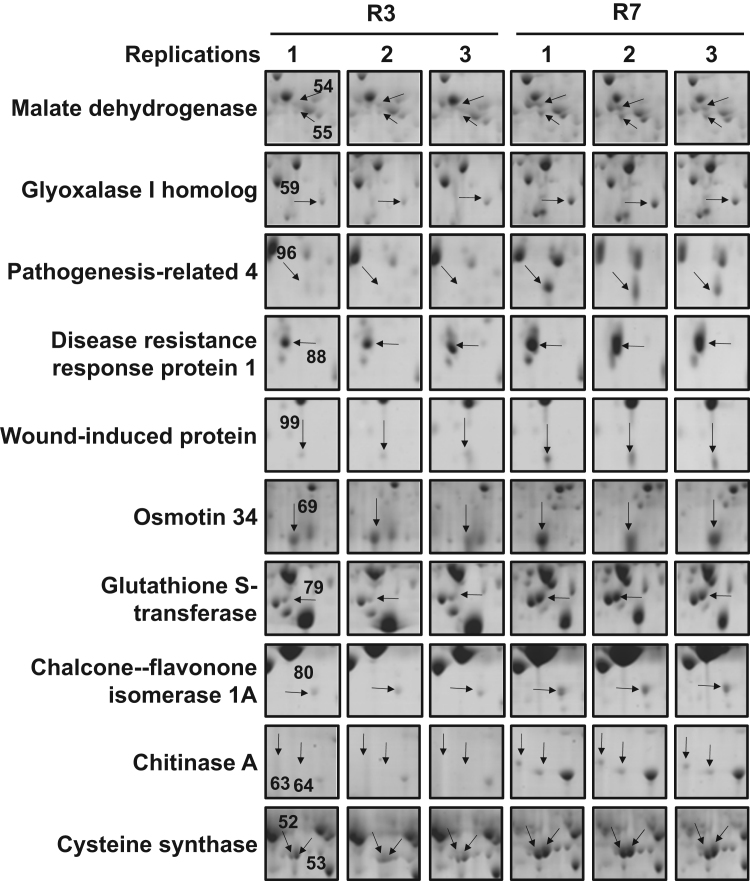
Zoom gel regions corresponding to Fig. 2 in Ref. [1] to get a better picture of differentially modulated protein spots.

**Fig. 2:**
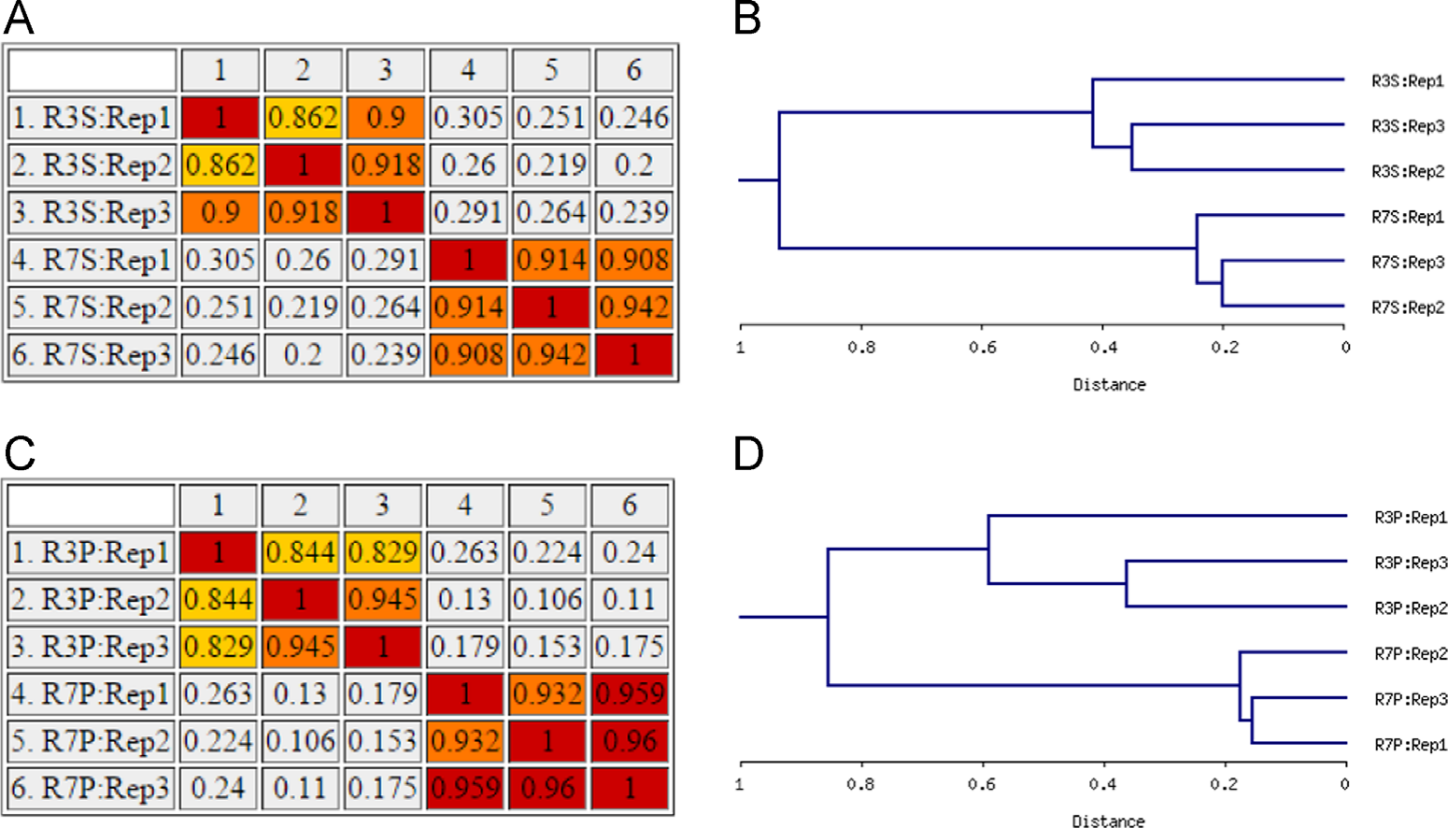
Analysis of correlation between the biological replicates of supernatant (A & B) and pellet (C & D) fractions. Correlation matrix (A & C) and dendrograms (B & D) were generated using the percentage volumes of differential protein spots of supernatant and pellet fractions using NIA array software.

**Fig. 3:**
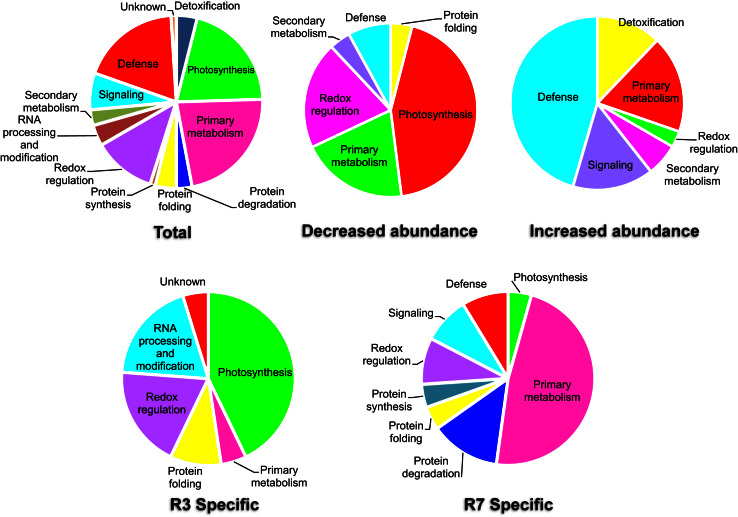
GO analysis of the identified differentially modulated spots from the 2-D gels of R3 and R7 leaves.

**Fig. 4:**
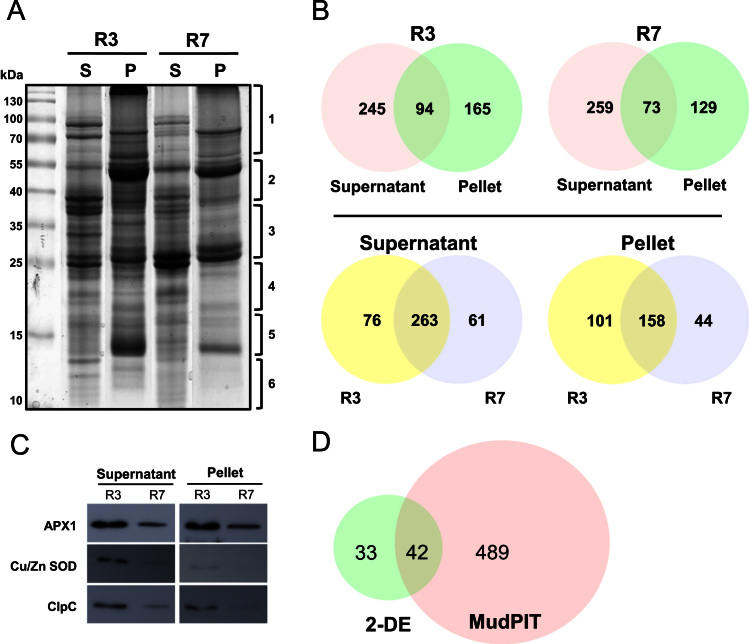
Shotgun proteome analysis of the senescence-related proteins. (A) SDS-PAGE profile of supernatant (S) and pellet (P) fractions of R3 and R7 stages, obtained after PEG fractionation. (B) Venn diagrams depicting the distribution of the proteins in the supernatant and pellet fractions of R3 and R7 stages. (C) Western blot analysis for the validation of the MS-identified proteins using ascorbate peroxidase1 (APX1), Cu/Zn-superoxide dismutase (Cu/Zn-SOD) and ClpC (HSP100) antibodies. (D) Venn diagram showing the distribution of the identified proteins in the 2-DE and shotgun proteomic approaches.

Figures reported here depict the data (Fig. 1), statistical analysis (Fig. 2), functional annotation (Fig. 3), and comparative analysis (Fig. 4) of the identified proteins from R3 and R7 leaves. Supplementary Tables show the list of differential modulated spots (Supplementary Tables 1 and 2), proteins identified by 2-DE MS (Supplementary Tables 3 and 4), and shotgun proteomics (Supplementary Tables 5 and 6) approaches, from PEG –supernatant and –pellet fractions of R3 and R7 leaves. Detailed description of the data and methods is reported previously [1].

## 2 Experimental design, materials and methods

### 2.1 Plant material

R3 and R7 leaves were collected from the soybean plants grown at the experimental field of the Department of Functional Crop, National Institute of Crop Science (NICS), Rural Development Administration (RDA) at Miryang, South Korea (latitude 35N) in June.

### 2.2 Protein isolation

For the identification of senescence-associated proteins, first, total leaf proteins were extracted in 10 mL of Tris-Mg-NP-40 buffer (0.5 M Tris–HCl, pH 8.3, 2% v/v NP-40, 20 mM MgCl_2_) followed by their precipitation using 15% PEG, as described previously [1]. Proteins from PEG-supernatant and -pellet fractions were then recovered using methanolic-phenol ammonium acetate precipitation method.

### 2.3 Two-dimensional gel electrophoresis, MALDI-TOF/TOF MS and shotgun proteomic analysis

Two-dimensional gel electrophoresis (2-DE) and MALDI-TOF/TOF MS identification were carried out as described in detail previously [1], [2]. In brief, protein pellets after methanolic–phenol ammonium acetate precipitation were dissolved in rehydration buffer containing 7M Urea, 2M thiourea, 4% v/v CHAPS, 2M DTT, and 0.5% v/v IPG buffer pH 4–7 (GE Healthcare, Waukesha, WI, USA) and quantified by 2D-Quant kit (GE Healthcare). A total of 600 µg of proteins from three biological replicates of PEG-supernatant and –pellet fractions of R3 and R7 leaves, were loaded on the 24 cm IPG strips (pH 4–7) and first and second dimension separation of proteins was carried out as described previously [1]. Colloidal Coomassie Brilliant Blue (CBB) stained gels were scanned using a transmissive scanner (PowerLook 1120, UMAX) and analyzed by ImageMaster2DPlatinum software (ver. 6.0, GE Healthcare). Student׳s *t*-test was used to determine the statistically significant differentially modulated spots between the 2-DE gels of R3 and R7 leaves (*p*<0.05) [1]. Spots showing differential modulation were excised from the gels, destained, subjected to in-gel digestion and identified by a MALDI-TOF/TOF-MS (ABI 4800, Applied Biosystems, Framingham, MA, USA) as described in detail previously [1], [3]. Shotgun proteomic analysis of R3 and R7 leaf proteins was carried out using QExactive^TM^ Orbitrap High-Resolution Mass Spectrometer as described previously [1].

### 2.4 Statistical analysis and functional annotation of the identified proteins

Identified proteins were functionally annotated using Gene Ontology (GO) database. Correlation matrix and dendrograms were generated using the percentage volumes of differential protein spots of supernatant and pellet fractions from three biological replicates using NIA array software [4].
