# Supplementary material for: Proteome data associated with the leaf senescence in Glycine max
Source: Data Brief. 2016 Aug 28;9:90–5. doi: 10.1016/j.dib.2016.08.045 (PMC5013252; doi:10.1016/j.dib.2016.08.045)
Supplement: Supplementary file 2 — Supplementary material [file mmc2.docx]

**Conflict of interest**

The authors have declared that no competing interests exist.
